# Does service integration improve technical quality of care in low-resource settings? An evaluation of a model integrating HIV care into family planning services in Kenya

**DOI:** 10.1093/heapol/czx090

**Published:** 2017-11-24

**Authors:** Richard Mutemwa, Susannah H Mayhew, Charlotte E Warren, Timothy Abuya, Charity Ndwiga, Jackline Kivunaga

**Affiliations:** 1 Department of Global Health & Development, London School of Hygiene & Tropical Medicine, 15-17 Tavistock Place, London, WC1H 9SH, UK; 2 Population Council, 4301 Connecticut Avenue NW, Suite 280 Washington, DC 20008 United States; 3 Population Council, Ralph Bunche Rd, Upper Hill, Nairobi, Kenya

**Keywords:** Service integration, technical quality of care, HIV, family planning

## Abstract

The aim of this study was to investigate association between HIV and family planning integration and technical quality of care. The study focused on technical quality of client–provider consultation sessions. The cross-sectional study observed 366 client–provider consultation sessions and interviewed 37 health care providers in 12 public health facilities in Kenya. Multilevel random intercept and linear regression models were fitted to the matched data to investigate relationships between service integration and technical quality of care as well as associations between facility-level structural and provider factors and technical quality of care. A sensitivity analysis was performed to test for hidden bias. After adjusting for facility-level structural factors, HIV/family planning integration was found to have significant positive effect on technical quality of the consultation session, with average treatment effect 0.44 (95% CI: 0.63–0.82). Three of the 12 structural factors were significantly positively associated with technical quality of consultation session including: availability of family planning commodities (9.64; 95% CI: 5.07–14.21), adequate infrastructure (5.29; 95% CI: 2.89–7.69) and reagents (1.48; 95% CI: 1.02–1.93). Three of the nine provider factors were significantly positively associated with technical quality of consultation session: appropriate provider clinical knowledge (3.14; 95% CI: 1.92–4.36), job satisfaction (2.02; 95% CI: 1.21–2.83) and supervision (1.01; 95% CI: 0.35–1.68), while workload (−0.88; 95% CI: −1.75 to − 0.01) was negatively associated. Technical quality of the client–provider consultation session was also determined by duration of the consultation and type of clinic visit and appeared to depend on whether the clinic visit occurred early or later in the week. Integration of HIV care into family planning services can improve the technical quality of client–provider consultation sessions as measured by both health facility structural and provider factors.

Key Messages

- Integration of HIV care into family planning services can improve the technical quality of care as measured by both facility structural and provider factors. However, provider factors have bigger impact on the effect of integration on technical quality compared with structural factors.
- The association between service integration and technical quality of care works through resultant changes in particular elements of the client–provider consultation session, namely: duration of the consultation, and type of clinic visit; with the weekly scheduling of client clinical visits also playing a significant role.
- Good technical quality of the client–provider consultation can never be assured in the context of: inadequate FP commodities and reagents, weak infrastructure, low provider clinical knowledge, poor supervision and technical support systems, and demotivated staff.

## Introduction

Integration of HIV into reproductive health services (RH), including family planning (FP), is now seen as an important component of successful achievement of, in particular, explicitly health-related Sustainable Development Goal 3, of ensuring ‘…healthy lives and promote well-being for all at all ages’ especially in low- and middle-income countries (UNDP 2015; United Nations 2016; Warren *et al.* 2017a). A central argument for integration of HIV services into FP services in primary-level health facilities is that it has the potential to improve uptake of FP, HIV services or both (Yoder and Amare 2008; Grossman *et al.* 2013; Wilcher *etal*. 2013; Kimani *et al.* 2015a; Cohen *et al.* 2017). A widely held assumption is that integration enhances quality of clinical care, a condition that both stimulates and consolidates demand for services (Spaulding *et al.* 2009; Pfeiffer *et al.* 2010; Herrel *et al.* 2016; Church *et al.* 2017; Warren *et al.* 2017b). However, other perspectives and experiences with integration present a contrary view and offer cautionary misgivings (for instance, Foreit *et al* 2002; Hwang *et al* 2013; Stephenson *et al* 2015). Thus, more empirical evidence is needed to test this association. This study aimed to investigate association between service integration and quality of care and the nature of that association, focussing exclusively on integration of HIV care into FP services.

As opposed to vertically provided care, integrated care refers to the mode where clients may receive more than one service at the point of care (Kodner and Spreeuwenberg 2002; Church and Mayhew 2009; Stephenson *et al* 2015; Banfield *et al* 2017). The literature is not consistent in the taxonomies or definitions applied to identify different types of integration (for instance, Shortell *et al* 2000; Kodner and Spreeuwenberg 2002; Hwang *et al* 2013; Banfield *et al.* 2017). In this study, we refer to ‘functional integration’ in the sense used by the Integra project: that a facility is able to offer multiple services (HIV-related services and FP-related services) to a client during a single visit (see Mayhew *et al.* 2016). Functional integration can take different forms, most obviously at the provider level (where a client receives multiple services in a single client–provider consultation session), facility level (where a client receives multiple services from different providers or rooms during the same visit) or some combination of the two (Mutemwa *et al.* 2013).

The investigation focused on technical quality of care (Donabedian 1988; Blumenthal 1996; Evans *et al.* 2001; Øvretveit 2002), rather than on quality of care more broadly. Arguments for integration of HIV and reproductive health services in the literature point to increases in service uptake mainly due to: availability of an expanded and accessible range of services to the client per clinic visit, and improved clinical interaction between the client and provider (Foreit *et al.* 2002; Kaba and Alem 2006; Mullick *et al.* 2006; Liambila *et al.* 2008; Church and Mayhew 2009; Spaulding *et al.* 2009; Pfeiffer *et al.* 2010). Both these factors relate to technical quality of care (Donabedian 1988; Evans *et al.* 2001). The goal of this investigation was, therefore, to determine whether or not integration is associated with technical quality of care.

The study adopted a conceptual perspective on quality grounded in the Donabedian quality assessment framework (Donabedian 1966, 1988). The Donabedian framework prescribes a three-part approach to quality assessment comprising: ‘structure’, ‘process’ and ‘outcome’. Structure denotes the context of physical resources (staff numbers, equipment, amenities, drugs and administrative attributes) in the health facility where the process of care occurs. Process denotes actual provision and receipt of care that involves both clinical and interpersonal interaction between the provider and client. Outcome denotes the health status of the client and population directly attributable to the care received; and which may be physical, psychological or behavioural. Integration, by definition, is a ‘process’-centred strategy. Client experience of integrated services occurs during the process of care, within a context defined by structural attributes. This study focussed on ‘process’ and the investigation included examining the relationship between structural and provider elements and quality of care provided during that process. The investigation excluded ‘outcome’ mainly for scientific and practical reasons articulated by Donabedian (1966, 1988) and Evans *et al.* (2001). By design, the central question for this study was whether service integration has implications for quality of care, irrespective of the health outcome.

Finally, the clinical consultation session between client and provider in the facility was considered the most appropriate arena for investigating any relationship between integration and technical quality of care. Client–provider clinical consultations are the frontline of service provision in any health facility, the interface between facility and the local population (Rull and Tidy 2014). Clinical consultations provide the space for facility expressions of technical quality of care and bring into focus the clinical performance of providers in the facility, which is central to any quality of care assessment (Donabedian 1988). Thus, most crucially, clinical consultation sessions define the prospects for a future repeat clinic visit by the client, a future first visit by a new client and future health outcomes for the client.

## Methods

This article is based on a cross-sectional health services evaluation study, which was part of the *Integra* Initiative—a 5-year quasi-experimental intervention study, designed to assess the benefits and costs of integrating HIV and RH services in public health facilities in Kenya and Swaziland (Warren *et al.* 2013). The criteria included facility utilisation of > 50 infants per month receiving first immunisations at 6 weeks and not <100 FP clients per month; at least two FP providers qualified in and currently providing FP services; availability of FP, HIV and STI services. A detailed description of the protocol has been published (Warren *et al.* 2013). The cross-sectional sub-study reported here was implemented in the final year of Integra as a multi-method health facility assessment exercise in 12 public health facilities in Central Province, Kenya. Most recent reports indicate the total number of public health facilities of similar size in what was Central Province at the time of the study was 105 (Muchemi and Gichogo 2014); however, numbers have likely changed with the introduction of counties that are smaller than the past borders of provinces. Of the 12 study facilities, 6 facilities were allocated to the intervention arm, while the other 6 served as comparison facilities. According to the original Integra study design, intervention facilities implemented integrated services while comparison facilities provided standard non-integrated care (i.e. HIV care separately from FP services). This later changed following a shift in government policy, as explained in the Integra Index section below. By agreement with the Government of Kenya, initial clinical supplies were provided to all study facilities, after which routine government medical supply systems took over.

Health facility assessments were conducted at each study facility. These included facility audits (staff numbers, clinical supplies and amenities), provider interviews (clinical knowledge and practice) and client–provider interaction observations (consultation session process).

Facility audits were conducted using a facility-audit checklist. Provider interviews and client–provider interaction observations were conducted on non-random consecutive samples of health providers and client–provider consultation sessions. Provider interviews were conducted using a structured questionnaire with the next available and willing health worker in the study facility. Similarly, client–provider consultations were observed in each facility using a checklist, as long as both the client and provider consented. All interviews were conducted in English. Table 1 presents a summary of facilities assessed, providers interviewed and client–provider clinical consultations observed, by study arm.

**Table 1:** Number of facilities assessed, consultation sessions observed and providers interviewed, by study arm

Facility type	Health facilities	Provider respondents	Consultation sessions
Comparison arm	6	19	170
Integra intervention arm	6	18	196
Total	12	37	366

Ethical approval was granted for the study by authors institute. Each respondent provided written informed consent to participate in the study and be interviewed and/or observed.

### Integra index of service integration

During the Integra study, the government in Kenya formally adopted and accelerated implementation of integrated HIV and FP services in all public health facilities. This removed operational distinction in service provision between facilities initially allocated to intervention and comparison arms of the study. Consequently, assessment of the primary outcome was shifted from comparison of study arms to comparison of individual facilities depending on the level of integration achieved by each facility over the assessment period. A functional integration index was developed at the end of data collection in the Integra study, to measure the level of integration at a given point in time in each study facility independent of its initial assignment to comparison or intervention. This is described in detail elsewhere (Mayhew *et al.* 2016) but involves the development, using Bayesian techniques, of a facility- and time-specific score for each facility over time. Using a form of propensity-score analysis the functional Index uses four indicators to measure the degree of functional integration achieved by each study facility over time. These indicators are: % days in the week on which any RH services (defined as FP, post-natal care and ante-natal care) and any HIV-related services [defined as antiretroviral therapy (ART), cervical cancer screening, CD4 count services, HIV/AIDS testing services and STI treatment] are accessed; % clients who receive any RH services AND any HIV-related services in one of their provider contacts; % clients who receive any RH services AND any HIV-related services during their visit to the facility (1 day); location of ART and functionality of referral system to ART for SRH clients.

### Technical quality of care score and facility structural factors

The ‘technical quality of client–provider consultation sessions’ (TQCS) was the outcome and ‘integration’ as measured by the Integra Index as the exposure. Technical quality was defined as the degree to which a consultation session delivered on five service elements prescribed by the study-intervention clinical protocol: ‘initial greeting & assessment of client’; ‘client FP counselling & provision’; ‘STI risk assessment & condoms’; ‘HIV counselling & testing’ and a combined cluster of questions that included ‘gynaecological and breast examinations, pap smears, child health, and clinical recording’. The five elements were covered by 22 questions in a client–provider consultation observation checklist. The checklist was used by investigators to observe the 366 client–provider clinical sessions.

**Figure 1:**
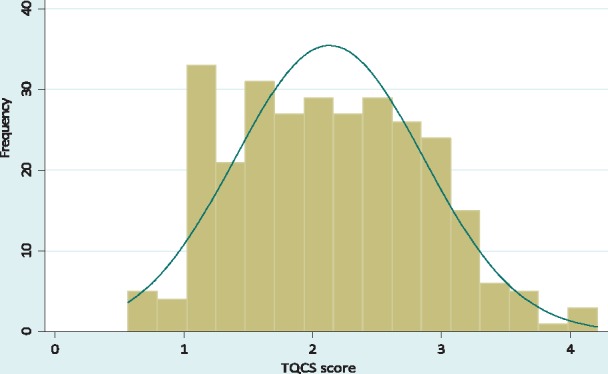
Normal distribution of the TQCS score

The 22 questions on the prescribed five service elements had responses coded as ‘1 = observed’ and ‘0 = not observed’. Any number of questions for a service element were totalled to a maximum score of 1. The service elements were equally weighted. Every observed consultation session was scored on each of the five service elements and the scores added to construct its TQCS score. A consultation session needed to score five points for a perfect TQCS score (range, 0–5). The final TQCS score was continuous, with a normal distribution and value-range of 0.5694–4.2115 (mean 2.1452; median 2.1368; SD 0.7288). Figure 1 illustrates the following.

Reliability of the TQCS score was tested using Cronbach’s alpha. Table 2 presents results of the Cronbach’s alpha test, suggesting significant reliability of the TQCS score: alpha coefficient *α = *0.7048; correlation with underlying factor *r = *0.8395. The item-rest results suggest that ‘initial greeting & assessment of client’ and ‘HIV counselling & testing’ may not fit that well with the rest of other elements in the score. However, their removal tests did not indicate significant improvement in the Cronbach’s alpha coefficient; thus, they were left in the score.

**Table 2:** Reliability of TQCS score using Cronbach’s alpha test

TQCS score reliability coefficient	0.7048			
Correlation with underlying factor	0.8395			
Service element	*n*	Item-test correlation	Item-rest correlation	Alpha (*α*)

Initial greeting and client assessment	366	0.6318	0.3982	0.6818
FP counselling and provision	366	0.6722	0.4536	0.6593
STI risk assessment and condoms	366	0.7736	0.6024	0.5952
HIV counselling and testing	366	0.6290	0.3944	0.6833
Four maternal and child health services	366	0.6793	0.4636	0.6551

Apart from the TQCS score, four other factors with potential impact on TQCS were used to characterise the client–provider consultation session: *consultation duration, day of the week, time of day*, and *main reason for clinic visit* (repeat FP visit or first-time FP visit) (Table 3, client–provider consultation session characteristics). These four characteristics of the consultation session were identified from previous experience, during data-collection observations and in a separate qualitative analysis of provider in-depth interviews that suggested variations in consultation practices according to these characteristics (Mutemwa *et al.* 2013). No previous study in the literature has systematically described or assessed characteristics of a client–provider consultation session that might impact its quality.

**Table 3:** Client–provider consultation session characteristics, facility-level structural and provider factors, and univariable associations with technical quality of care

Associated with TQCS (*α = 0.05*)		Not associated with TQCS (*α = 0.05*)	
Client–provider consultation session characteristics			
Consultation duration Day of the week Main reason client came to FP clinic		• Time of day of consultation session	
Facility-level structural and provider factors			
Structural FP commodities Drugs availability General clinic supplies Reagents Infrastructure Staff numbers in MCH	Provider Mentorship Communication between staff Supervision Job satisfaction Staff clinical knowledge Workload	Structural IEC and visual aids materials availability Clinical protocols, policies etc Clinical information system Total facility staff (*N*) Capitation (ratio) Catchment population	Provider Length of staff experience in public health Reported effective staff management Formal medical training

The covariate ‘day of the week’ initially covered the five working days of the week, excluding weekend days because these were not included in data collection and many facilities were closed on these days. The 5 days were subsequently collapsed to two categories representing early (Monday and Tuesday) and latter parts of the working week (Wednesday to Friday), based on the hypothesis that some facilities may be systematically scheduling client visits and services on specific days of the week, as suggested from previous experience elsewhere as well as published guidelines (Neamatalla and Verme 1995; Islam 2007; WHO 2008; Ditekemena *et al.* 2012; Kwambai *et al.* 2013). The question was whether or not any such existing scheduling had implications for technical quality of consultation sessions.

In addition, 21 facility-level structural and provider factors were identified for description of the facility context within which the consultation session occurred (Table 3, facility-level structural and provider factors). As in development of the TQCS score, each of the structural and provider factors was constructed into a continuous variable from a set of questions on more than one equally-weighted components and each facility scored on each factor. All client–provider consultation sessions observed in the same facility had the same scores on all the structural and provider factor attributes for that facility. The individual scales and distributions of the factor scores were like that of the TQCS score.

### Statistical analysis

#### Propensity score analysis and causal modelling

The unit of analysis was the clinical consultation session. Data from facility audits, provider interviews, and client–provider consultation sessions were treated as hierarchical. Client–provider consultation sessions were nested in providers who were in turn nested in facilities. However, as an artefact of the data-collection exercise, client–provider consultation sessions were not linked to provider interviews data. Therefore, all provider interview variables were converted to facility-level means and upgraded to structural factors, reducing the hierarchy to two levels.

All statistical analyses were performed in STATA 11.2 (STATACORP 2012). Because the client–provider consultations were not randomly sampled, propensity score analysis was used to correct for selection bias in the estimation of treatment effect (Rosenbaum and Rubin 1983; Rosenbaum 2005; Guo and Fraser 2010; Salzaberg 2012). To test for the presence of selection bias, association between the treatment-group variable and the four consultation-session covariates was assessed using independent sample t-test for ‘session duration’ and Pearson’s Chi-square test for the rest (see Table 4) (Rosenbaum 2005; Guo and Fraser 2010). Propensity scores were generated using logistic regression of the treatment variable on the two significantly associated covariates in Table 4, and greedy matching was performed to balance the data. A post-matching assessment of reduction in bias was performed.

**Table 4:** Treatment-group comparisons on covariates before and after propensity score matching and achieved reduction in bias

Before propensity score matching					After propensity score matching				Bias reduction
Covariate	Mean/prop. (π)	*t*-value/χ2	*P*-value	Bias	Mean/Prop. (π)	*t*-value/χ2	*P*-value	Bias	(%)
Consultation duration (minutes)									
Comparison	20.54	−1.87	0.062	3.85	21.41	−1.3	0.194	3.21	16.62
Intervention	24.39				24.62				
Day of the week									
Comparison	0.46	11.3	0.001	0.08	0.5	0	1	0	100
Intervention	0.54				0.5				
Time of the day									
Comparison	0.46	0.78	0.377	0.08	0.5	1.89	0.169	0	100
Intervention	0.54				0.5				
Main reason for clinic visit									
Comparison	0.46	6.81	0.009	0.08	0.5	0	1	0	100
Intervention	0.54				0.5				

*Prop. (π) = proportion; x^2^ = Chi-square*.

The original sample size for client–provider consultations was 366, with 170 in the comparison and 196 in the intervention arms. After propensity score matching, the sample size was reduced to 286 on resampling, with 143 sessions in each of the study arms. That represented a loss of about 22% on the original sample; however, the resampling produced a more balanced dataset with considerably reduced overt bias (Table 4).

**Figure 2:**
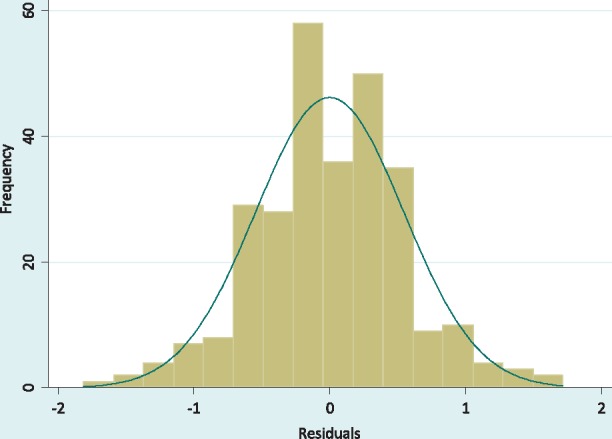
Multivariate normal distribution of the TQCS residuals

An intraclass correlation test for clustering by facility indicated a coefficient of 0.45, suggesting that a multilevel analysis incorporating facilities and facility characteristics would be appropriate. A random intercept model was fitted to estimate integration effect with integration index as a continuous generalised (all facilities) exposure variable, using restricted maximum likelihood to ensure less biased random-effects estimates, especially given the small number of level-2 units (facilities). A sensitivity analysis was then performed using Wilcoxon’s signed-rank test to determine the sensitivity of the effect of integration to hidden bias (Rosenbaum and Rubin 1985; Guo and Fraser 2010; Jordan 2012). The results for this analysis are presented in Table 5 and discussed in the Results section. Ten of the structural and provider covariates identified as significantly associated with TQCS in Table 3 were then added to the model to condition the effect of integration. ‘Communication’ and ‘staff numbers in mch’ were excluded from the model due to multicollinearity. This multivariable random intercept model was not significantly different from linear regression (Likelihood ratio test: *χ*^2^<0.001, *P *=* *1.00); therefore, a multivariable linear regression model was fitted to the data, with TQCS residuals assumed to follow a multivariate normal distribution (Figure 2). Table 6 presents results of the fitted final multivariable regression model, which had the general form of:

$$TQCS_{\text{i}}=\alpha +\beta_{\text{j}}INDEX_{\text{ji}}+\beta_{\text{k}}FACTOR_{\text{ki}}+\varepsilon_{\text{i}}$$

where TQCS_i_ is the technical quality score for a client–provider consultation session (*i*th consultation session); *α* is the technical quality score for a consultation session in a non-integrated facility with minimally effective to dysfunctional structural and provider factors; *β*_j_ is the increase in the technical quality score for a consultation session for a 1 unit increase in the integration index score of the host facility with all the structural and provider factors held constant; INDEX_ji_ is the integration index score for a facility; *β*_k_ is the increase in the technical quality score for a consultation session for a 1 unit increase in the structural or provider factor under consideration in the host facility, with the rest of the other factors and the facility integration score held constant; *FACTOR*_ki_ is the structural or provider factor under consideration in the multivariable model—‘please note that the term β_k_FACTOR_ki_ represents all the 10 covariates that were entered in the multivariable model but which cannot be individually listed in the model equation above for reasons of space and clarity’; ε_i_ represents TQCS residuals.

**Table 5:** Sensitivity analysis for the effect of Integration on TQCS: range of significance levels for the Wilcoxon’s signed-rank statistic

Γ (Gamma)	Minimum *P*-value	Maximum *P*-value
1	<0.001	<0.001
1.3	<0.001	<0.001
1.9	0	0.005
2	0	0.010
2.3	0	0.043
2.4	0	0.063
3	0	0.290

* Not all gamma values in the used range at analysis are presented here; however, no gamma value is left out between Γ = 2.3 and Γ = 2.4.

**Table 6:** Association between Integration and TQCS, accounting for structural and provider factors

Covariate	Coefficient	SE	[95% CI]
Integration (index)	0.44	0.193	0.63, 0.82*
Structural factors			
FP commodities	9.64	2.323	5.07, 14.21**
Drugs	0.80	1.090	−1.35, 2.95^§^
Reagents	1.48	0.229	1.02, 1.93**
General clinical supplies	−3.64	1.766	−7.12, -0.17*
Infrastructure	5.29	1.221	2.89, 7.69**
Provider factors			
Supervision	1.01	0.338	0.35, 1.68*
Job satisfaction	2.02	0.411	1.21, 2.83**
Staff clinical knowledge	3.14	0.608	1.95, 4.33**
Workload	−0.88	0.442	−1.75, -0.01*
Mentorship	−0.01	0.171	−0.35, 0.32^§^
*R^2^* = 0.45			

* *P < 0.*05,

** *P* < 0.001,

§ *P* > 0.1.

The model was repeatedly fitted to test several interactions between integration index and the facility provider and structural factors. In addition, several counterfactuals were investigated, using statistical methods recommended by King *et al* (2000), to determine the impact of HIV-into-FP integration on technical quality of the client–provider consultation session under different counterfactual scenarios of structural and provider factors, each of the clusters of structural and provider factors was alternately either fixed at the mean-values of its respective factor-scores or the factor scores were allowed to vary across observations. Fixing factor scores to the mean-values represents setting the factor to a minimum acceptable/good standard and not any lower, while varying the scores exposes interaction with the whole range of low to high factor scores.

## Results

### Association between functional integration & TQCS

The study found a positive association between service integration and TQCS, which remained after controlling for the 10 significant facility-level structural and provider factors (Table 6). A sensitivity analysis showed that the study is somewhat sensitive to hidden bias and would be altered, at Γ > 2.3 (Table 5). That is, to attribute the observed positive association between integration and TQCS to a (unmeasured hidden) factor other than integration, the unobserved covariate would have to increase the odds of exposure by more than a factor of Γ = 2.3. This Γ value is fairly high meaning hidden bias is unlikely and thus, based on this study, it appears that there is positive causality between integration and TQCS; and the evidence is sufficiently robust against hidden bias.

### Accounting for structural and provider factors

Except for 2 covariates (‘general clinical supplies’; ‘mentorship’), the rest of the factors presented direction of association with TQCS as expected intuitively and from published evidence (Table 6). In multivariable analysis, ‘availability of general clinical supplies’ indicated a negative association with TQCS (−3.64, *P* < 0.05). A possible logical explanation for the negative association is presented under Discussion section. ‘Mentorship’ also indicated a negative association albeit considerably small in magnitude with no evidence (−0.01, *P* > 0.1), after accounting for the other factors. ‘Availability of drugs’ was the only other covariate with no evidence for association with TQCS (0.80, *P* > 0.1), conditional on the other factors.

The structural factor with strongest positive association with TQCS, after controlling for other factors, was ‘(availability of) FP commodities’ (9.64, *P* < 0.001), followed by ‘(adequate) infrastructure’ (5.29, *P* < 0.001), and the ‘reagents’ (1.48, *P* < 0.001). Whereas the provider factor with the strongest positive association with TQCS was ‘staff clinical knowledge’ (3.14, *P* < 0.001), followed by ‘job satisfaction’ (2.02, *P* < 0.001). ‘Supervision’ indicated the lowest effect on TQCS among factors with strong evidence for positive association with TQCS (1.01, *P* < 0.05), after accounting for the other factors. As expected ‘workload’ indicated strong evidence for negative association with TQCS (−0.88, *P* < 0.05), after accounting for the other factors.

**Figure 3:**
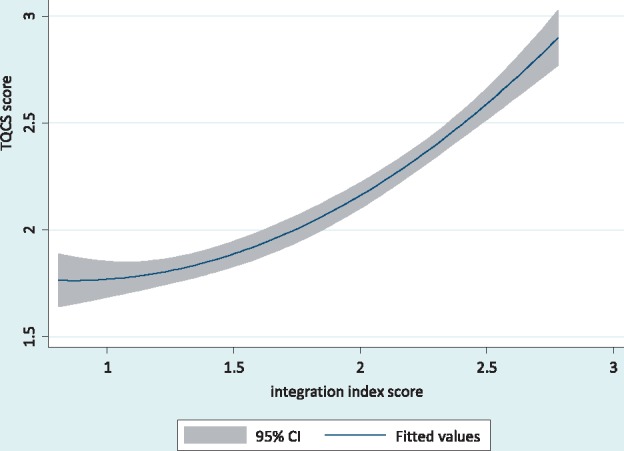
Fitted values for technical quality of client–provider consultations with confidence intervals

Figure 3 is a plot of fitted values for technical quality with their CIs, demonstrating a direct association between the degree of integration measured on the integration index and technical quality of the client–provider consultation session measured on the TQCS score. The relationship appears non-linear and monotonic.

Table 7 presents results from counterfactual analysis, with four counterfactual scenarios of structural and provider factors and how they impact the effect of HIV-into-FP integration on TQCS. The factors included in the analysis were restricted to the list reported in Table 6 as these were the ones found to be important enough to cause concern. Ensuring that all the 4 provider factors in Table 6 were in good operational standard in the health facility (fixed at good standard) seems to predict the most impact on the effect of integration on TQCS (integration effect: 0.86 and 0.57), whether or not the structural factors were left to vary. On the other hand, ensuring that the five structural factors were in good operational standard in the facility seems to predict much lower impact on the effect of integration on TQCS, especially when provider factors were left to vary (integration effect: 0.42). These counterfactual results represent different combinations of the status of available capacity in a facility and how that may impact on the extent to which integration improves the technical quality of client–provider consultation sessions. Clearly, provider factors, if strengthened in the facility, seem to promise a much better effect of integration on technical quality of the consultation session compared with structural factors, whether the capacity of structural factors is equally strong or is weak. This has a practical policy implication for especially resource-poor low- and middle-income country health systems, as highlighted under the discussion session below.

**Table 7:** Impact of HIV-into-FP integration on technical quality of care under different counterfactual scenarios of structural and provider factors

Effect of integration	Provider factors	Structural factors
0.86 [0.51, 1.22]	Fixed at good standard	Varying
0.42 [0.22, 0.63]	Varying	Fixed at good standard
0.44 [0.63, 0.82]	Varying	Varying
0.57 [0.43, 0.70]	Fixed at good standard	Fixed at good standard

None of the fitted interactions between integration index and provider and structural factors were significant enough to warrant further investigation and reporting.

### Client–provider consultation session characteristics, structural and provider factors and TQCS

Of the four identified consultation session characteristics (Table 3A), three demonstrated significant association with TQCS in univariable regression analyses (not shown: ‘consultation duration’, crude Coef.=0.011 *P* < 0.001; ‘day of the week’, crude Coef.=0.183 *P* < 0.001; ‘main reason for clinic visit’, crude Coef.=−0.497, *P* < 0.001). The technical quality of a client–provider consultation seemed to increase with its duration; the mean consultation duration was 22 min (median = 16 min), range: 2–160 min. Consultations in early part of the week appeared to be of lower quality compared with those offered in latter part of the week. Clients who attended the clinic on FP repeat visits or FP-method review received lower technical quality consultations than those attending for the first time, were switching a contraceptive method or coming back after a long gap without contraception. There was no evidence of difference in TQCS between consultations held in the mornings and those held in the afternoons (not shown: crude Coef. = 0.097, *P* = 0.228).

Because ‘day of the week’ is a scheduling factor, rather than a direct feature of the consultation process, its association with TQCS was tested for whether or not it depended on the distributions of ‘consultation duration’ and ‘main reason for clinic visit’ over the 5-day week. There was no significant evidence of association with either ‘consultation duration’ (*P* = 0.527), or ‘main reason for clinic visit’ (*P* = 0.369).

Of the 21 facility-level structural and provider factors, there was little or no evidence for association with TQCS for half (7) of the structural factors and two of the provider factors in univariable regression analyses (Table 3B). Three structural factors presented associations of infinitesimal magnitude (not shown: ‘total number of staff in the facility’, crude Coef.=0.002; ‘facility’s catchment population’, crude Coef. <0.001; ‘capitation’, crude Coef.<0.001); while the remaining four structural and two provider factors presented statistically non-significant associations (not shown: ‘availability of IEC& visual aids’, *P* = 0.832; ‘availability of clinical policies & protocols’, *P* = 0.173; ‘operational clinical information system’, *P* = 0.113; ‘formal clinical/medical training’, *P* = 0.283; ‘length of staff experience in public health’, *P* = 0.918; ‘reported effective staff management’, *P* = 0.869).The six structural and four provider factors that demonstrated significant univariable association with TQCS are discussed below in results from multivariable regression modelling.

## Discussion

This study set out to investigate the association between integration and technical quality of care in public health facilities providing integrated HIV and FP services in Kenya. The focus on client–provider consultations was essential not only because these sessions constitute the frontline of service provision in public health facilities, but they also provide the most appropriate arena for investigating technical quality of care for the integrated service model. The study was able to provide quantification of the effect of consultation characteristics suggested by previous qualitative analysis (Mutemwa *et al.* 2013). Our results have provided strong evidence for positive association between integration (as measured by the degree of integration achieved by each facility over the study period) and technical quality of client–provider consultations. The integration-quality nexus has been a central message in earlier empirical literature (Hwang *et al.* 2013; Herrel *et al.* 2017). Yet if the underlying ‘integration-impacts-quality-impacts-uptake’ assumption holds, this study adds to evidence reported by previous studies in Kenya such as by Grossman *et al* (2013) and Cohen *et al* (2017) both of which reported positive impact of integration on uptake of HIV care. Indeed, other analyses from our Integra study confirm such ultimate impact on uptake (Kimani *et al.* 2015a,b; Church *et al.* 2017).

### Technical quality and characteristics of client–provider consultation sessions

This is the first study in published literature to investigate associations between technical quality of the client–provider consultation and its four identified features identified through previous qualitative work: ‘consultation duration’, ‘main reason for clinic visit’, ‘day of the week’ and ‘time of day of consultation session’. With the caveat that these are crude associations, the first three demonstrated evidence for significant association with technical quality of the consultation. However, practical interpretation of these findings needs caution, well informed by clinical context. For instance, for positive association between ‘*consultation duration*’ and technical quality suggests that increasing the duration of consultations will increase technical quality. Previous studies have found that shorter consultation times fell below the minimum recommended session times for HIV care, directly impacting technical quality (Kim *et al.* 1998; Wilson and Childs 2002; Elmore *et al.* 2016). In practice, facility managers and providers need to pay attention to the reference baseline-duration and how far that duration may be increased without engendering challenges in other aspects of care. Consultation duration is particularly pertinent as it determines how much of the recommended clinical protocol is delivered to the client in each visit. For instance, a short duration means that the provider can deliver only a limited number of recommended service elements to the client or that minimum advised thresholds for certain elements are not met (Kim *et al.* 1998; Wilson and Childs 2002). Nevertheless, this evidence is not consistent across the landscape. A previous study reported negative perception, by providers, of increased waiting times for clients in facilities due to increased consultation duration per client resulting from service integration (Mutemwa *et al.* 2013). Indeed, a study by Lemon and Smith (2014) found ‘consultation content not consultation length improves patient satisfaction’, a finding corroborated in the mixed results of the more recent study by Elmore *et al* (2016). It appears therefore, in the case of consultation duration, that client as well as provider perspectives may play a critical role in getting consultation-duration adjustments right.

That clients who attended for repeat FP visits generally received lower technical quality than those attending for the first time or switching a contraceptive method, also needs cautious practical response. It may be that these two types of clients still receive the best technical quality of care for their type of visit. Considering the five elements of technical quality introduced earlier, in practice the protocol for repeat visits may be different in its delivered content compared with that for first visits. For instance, repeat clients may not need full description of all available contraceptive methods and/or they may not need a full health or HIV/AIDS education session. Yet, as Kim *et al.* (1998) observed in another study in Kenya, treating repeat client visits as brief routines potentially misses the opportunity to explore the client’s experience and satisfaction with the current treatment/intervention regimen and minimise the likelihood of clients dropping out or FP discontinuation. In an integration context short routine repeat visit consultations also risk missing opportunities for exploring and identifying emerging related problems such as the risk of HIV acquisition and the need for regular testing – indeed other analysis confirms that functional integration (with its longer consultation times) is also positively associated with regular HIV testing. Every consultation whether a first-visit or repeat, should be treated equally by providers as an opportunity and a decision point, though the content may be different. Future studies should consider treating the two types of visits as separate.

‘Day of the week’ demonstrated significant association with technical quality of client–provider consultations: those in the earlier part of the week appeared to be of lower quality compared with consultations offered later in the week. The initial hypothesis was that this association may be driven by the possibility that consultations are longer, on average, in the latter part of the week when often clinics are not busy and/or that most repeat clinic visits are scheduled for earlier days of the week. However, no evidence for significant association was found between ‘Day of the week’ and these two factors. Future investigations should examine the association between ‘Day of the week’ and each of the five individual dimensions of TQCS (‘initial greeting & assessment of the client’; ‘client counselling on FP’; ‘STI risk assessment & condoms’; ‘HIV counselling & testing’; and ‘other non-FP-related health issues’), to determine how these univariable associations are distributed over the 5-day week and the respective driving factors including the role of health system and facility contexts. Variations between facilities would also need to be explored to assess possible links to the distribution of resources over the week, such as availability of human resources each day and how that may influence TQCS.

Overall, findings on the characteristics of client–provider consultations point to the need for clinicians and facility managers to pay attention to the elements of the consultation session to improve its technical quality. The consultation-session attributes and elements should be considered both individually and jointly for their impact on technical quality of the care delivered.

### Technical quality and facility-level structural and provider factors

Facility-level structural and provider factors define the context within which client–provider consultations thrive or dysfunction. ‘Availability of general clinical supplies’ showed unexpected strong evidence of negative association with technical quality of the consultation session. This may be because ‘general clinical supplies’ include supplies for clinical services in the facility other than integrated HIV and FP, which may lead to intensification of clinical activity elsewhere in the facility, not benefitted HIV-FP services. However, this structural factor requires more empirical understanding and should be considered in future similar studies. A similarly surprising negative-association result was observed for ‘mentorship’, particularly that mentorship was one of the centrepieces of the integration intervention. But given the small size of the effect and the poor evidence indicated for it, not much can be said about that except to suggest the result may be due to chance or a mere statistical artefact. Further investigation is warranted.

The four structural and three provider factors that indicated positive association with technical quality of the client–provider consultation in Table 6 suggest the need for an adequately prepared facility context if good technical quality is to be assured and integrated care to succeed. Facility- and provider-preparedness as a necessary pre-condition to successful integration, and the need for a facilitative broader health system context, have been extensively discussed in previous studies in the literature (Hardee and Yount 1996; Oliff *et al.* 2003; Stone-Jimenez *et al.* 2010; Dudley and Garner 2011; Mutemwa *et al.* 2013). Our study confirms that good technical quality of the client–provider consultation can never be assured in the context of: inadequate FP commodities and reagents, weak infrastructure, low provider clinical knowledge, poor supervision and technical support systems, and demotivated staff.

Within the bounds of this study, the counterfactual scenarios in Table 7 tell a ‘poor man’s choices’ that ought to be examined with caution. Provider factors seem to have bigger impact on effect of integration on TQCS compared with structural factors. For resource-poor health systems, this presents an opportunity to rationalise available resources and decide how to allocate them to realise at least minimum benefit from implementation of integration. It would seem that in poor health systems where health facilities struggle with dysfunctional delivery infrastructure and weak human resource capacity, prioritising strengthening of the latter is logical. However, structural and provider capacity issues are not mutually exclusive; for instance, the condition of structural capacity within the health facility will affect provider motivation and ability to deliver a decent service to the patient—factors that are explored in Mayhew *et al.* (2017) in this issue.

We also found a strong suggestion that the more significant influence of provider factors compared with structural factors on effect of integration could be an artefact of how the TQCS score was constructed. All the 5 elements used to construct the score emphasise the role of the provider more explicitly than that of structural issues in the facility; this may be the inadvertent reason why provider factors appear to have more influence. To test this observation, one recommendation is that in future TQCS studies the conceptual definition of technical quality and the construction TQCS and its score be broadened to explicitly include both structural and provider factors in balanced measure, then subjected to rigorous analyses. Nevertheless, the other Integra paper in this issue (Mayhew *etal*. 2017) shows that providers (motivated to work in teams to support each other) can overcome structural barriers to delivering integrated care suggesting that provider factors may well have more impact on integration than structural factors.

In the end, for 11 of the 21 structural and provider factors excluded from the multivariable regression model (apart from the two factors excluded due to multicollinearity there was no evidence for association with technical quality of the consultation session in the univariable analyses. Further investigation is needed to ascertain why this may be the case for each one of the factors. However, it is worth noting that, in the case of ‘formal medical training’ and ‘length of staff experience in public health’ it has been known from previous studies that what may be most responsible for differentials in provider performance is not necessarily the formal medical school education or the nominal number of years of experience in practice. Rather, differentials in provider performance are likely due to individual provider knowledge-levels as determined by both medical training and, especially, quality of accumulated experience in clinical practice (Schulz *et al.* 1994; Curtis *et al.* 1995a,b; Kitahata *et al.* 1996; Holmes 1997). That may explain why the two factors above individually demonstrated no evidence of association with technical quality, while ‘staff clinical knowledge’ did. It is, therefore, recommended that health facility managers develop both recruitment and staff development strategies that ensure clinically knowledgeable provider-teams with the appropriate range of experiential skills. Further, previous qualitative work (Mutemwa *et al.* 2013) highlights the critical importance of the ability of providers working on complex service delivery to be able to communicate well, share workloads and support each other in teams if integrated care is to be delivered.

### Limitations of the study

This study has brought out useful insights into the understanding of technical quality of care in the context of integration of HIV and FP in a low-resource setting. However, a few quick observations need highlighting to bound the study findings and begin to pose questions about the context of application.

First, the study is specific to the HIV/FP model of integration. It may not be generalised with any certainty what findings the evaluation framework would produce in other service integration models; for instance HIV and postnatal care, or HIV and cervical cancer. Changing context to another integrated service model changes clinical protocols, provider profiles, client-profiles, cascades of care, structure of consultation sessions, and even regulatory frameworks. All these variables may shape both subtle and explicit elements of standards of care and hence technical quality. Thus, more studies of technical quality from the perspective of consultations are encouraged to generate more knowledge in the subject area.

Second, functional integration can take different forms: provider-level, unit-level, or a mix of both (Mutemwa *et al.* 2013). This study did not attempt to analyse for differentials between the integration formats in the way they might be individually associated with technical quality of care. The findings of this study should be understood with that in mind. This sub-area presents an additional opportunity for future research. It may also be possible to consider these findings for contexts of non-integrated services, particularly given that the client–provider consultation session is a universal feature of any health care system integrated or not.

## Conclusion

This study suggests that integration of HIV care into FP services can improve the technical quality of client–provider consultation sessions, and may therefore lead to better technical quality of care within developing health systems such as that of Kenya. The study has also demonstrated that the association between service integration and technical quality of care works through resultant changes in specific elements of the client–provider consultation session, particularly duration of the consultation and type of clinic visit, with the weekly scheduling of client clinical visits also playing a significant role.

However, any desired improvement in technical quality of care is conditional on the operational status of health facility structural and provider factors. Clinical commodities, laboratory supplies, and well-trained and appropriately-experienced staff should all be sufficient to meet service provision requirements; infrastructure should be adequate, staff should be adequately supervised and motivated.

## Ethical approval

Ethical approval was granted for the study locally in Kenya by the Kenya Medical Research Institute (Reference: NON/SSC/113); Population Council (IRB #443); as well as by the London School of Hygiene & Tropical Medicine Ethics Committee (Reference: 5426). Each respondent provided written informed consent to participate in the study and be interviewed and/or observed.

## Integra Initiative

The Integra Initiative team members are: At the London School of Hygiene & Tropical Medicine: Susannah Mayhew (PI), Anna Vassall (co-PI), Isolde Birdthistle, Kathryn Church, Richard Mutemwa, Manuela Colombini, Martine Collumbien, Natalie Friend-DuPreez, Natasha Howard, Joelle Mak, Dayo Obure, Sedona Sweeney, Charlotte Watts. At the Population Council: Charlotte Warren (PI), Timothy Abuya, Ian Askew, Joshua Kikuvi, James Kimani, Jackline Kivunaga, Brian Mdawida, Charity Ndwiga, Erick Oweya. At the International Planned Parenthood Federation: Jonathan Hopkins (PI), Lawrence Oteba, Lucy Stackpool-Moore, Ale Trossero; at FLAS: Zelda Nhlabatsi, Dudu Simelane; at FHOK: Esther Muketo; at FPAM: Mathias Chatuluka.
